# Supplementary material for: Computation for Latent Variable Model Estimation: A Unified Stochastic Proximal Framework
Source: Psychometrika. 2022 May 7;87(4):1473–502. doi: 10.1007/s11336-022-09863-9 (PMC9636119; doi:10.1007/s11336-022-09863-9)
Supplement: Supplementary file 1 — (pdf 692 KB) [file 11336_2022_9863_MOESM1_ESM.pdf]

Supplement to 'Computation for Latent Variable Model Estimation: A Unified Stochastic Proximal Framework'

In this supplement, we provide proofs of theoretical results in the main manuscript. We define some notations:

- $F(\boldsymbol{\beta}) = h(\boldsymbol{\beta}) + g(\boldsymbol{\beta})$
- $\partial f(\boldsymbol{x}) = \left\{\boldsymbol{z} \in \mathbb{R}^p : f(\boldsymbol{y}) \geqslant f(\boldsymbol{x}) + \boldsymbol{z}^\top(\boldsymbol{y} - \boldsymbol{x}) + o(\|\boldsymbol{y} - \boldsymbol{x}\|) \text{ as } \boldsymbol{y} \to \boldsymbol{x}\right\}$
- $\mathbf{G}_{\boldsymbol{\beta}}(\boldsymbol{\xi}) = \partial H(\boldsymbol{\xi}, \boldsymbol{\beta})/\partial \boldsymbol{\beta}$
- $\boldsymbol{\beta}^+_\gamma(\boldsymbol{\xi}) = \underset{\boldsymbol{x}\in\mathcal{B}}{\operatorname{argmin}} \left\{\boldsymbol{G}_{\beta}(\boldsymbol{\xi})^\top(\boldsymbol{x} - \boldsymbol{\beta}) + \mathbf{g}(\boldsymbol{x}) + \frac{1}{2\gamma}\|\boldsymbol{x} - \boldsymbol{\beta}\|^2_{\mathbf{D}}\right\}$
- $\boldsymbol{U}_\gamma(\boldsymbol{\xi}; \boldsymbol{\beta}) = \frac{1}{\gamma}(\boldsymbol{\beta} - \boldsymbol{\beta}^+_\gamma(\boldsymbol{\xi}))$
- $\mathbb{E}(\cdot \mid \boldsymbol{\beta}) = \int \cdot \pi_{\boldsymbol{\beta}}(\boldsymbol{\xi}) d\boldsymbol{\xi}$, $\pi_{\boldsymbol{\beta}}(\boldsymbol{\xi})$ is the posterior density for $\boldsymbol{\xi}$ given $\boldsymbol{y}$ and $\boldsymbol{\beta}$
- $\mathcal{F}_{t-1} = \sigma(\boldsymbol{\beta}^{(0)}, \boldsymbol{\xi}^{(k)}, 0 \leqslant k \leqslant t-1)$ is a filtration of σ-field
- $\mathcal{C}(\mathbb{R}^+, \mathbb{R}^p)$ denotes the continuous functions from $\mathbb{R}^+$ to $\mathbb{R}^p$
- $\operatorname{Prox}^{\mathbf{D}}_{\gamma,g}(\boldsymbol{\beta}) = \underset{\mathbf{x}\in\mathbb{R}^n}{\arg\min} \left\{g(\mathbf{x}) + \frac{1}{2\gamma}\|\mathbf{x} - \boldsymbol{\beta}\|^2_{\mathbf{D}}\right\}$

A Proof of Lemma 1

Our stochastic updates can be re-formated as

$$\boldsymbol{\beta}^{(t)} = \boldsymbol{\beta}^{(t-1)} - \gamma_t \boldsymbol{U}_{\gamma_t}(\boldsymbol{\xi}^{(t)}; \boldsymbol{\beta}^{(t-1)}). \tag{A.1}$$

Let $\boldsymbol{U}(\boldsymbol{\xi};\boldsymbol{\beta}) = \partial H(\boldsymbol{\xi},\boldsymbol{\beta})/\partial\boldsymbol{\beta} + \partial g(\boldsymbol{\beta})$ and $\boldsymbol{\epsilon}_\gamma(\boldsymbol{\xi};\boldsymbol{\beta}) = \boldsymbol{U}_\gamma(\boldsymbol{\xi};\boldsymbol{\beta}) - \mathbb{E}[\boldsymbol{U}_\gamma(\boldsymbol{\xi};\boldsymbol{\beta}) \mid \boldsymbol{\beta}]$. By Lemma 7 of Duchi & Ruan (2018), for $\boldsymbol{\beta} \in \mathcal{B}$ and $\epsilon > 0$,

$$\begin{aligned}
\|\boldsymbol{U}_\gamma(\boldsymbol{\xi};\boldsymbol{\beta})\| &\leqslant \|\boldsymbol{U}(\boldsymbol{\xi};\boldsymbol{\beta})\|, \text{ and} \\
\mathbb{E}[\|\boldsymbol{\epsilon}_\gamma(\boldsymbol{\xi};\boldsymbol{\beta})\|^2 \mid \boldsymbol{\beta}] &\leqslant \mathbb{E}[\|\boldsymbol{U}_\gamma(\boldsymbol{\xi};\boldsymbol{\beta})]\|^2 \mid \boldsymbol{\beta}] \\
&\leqslant \mathbb{E}[L_\epsilon^2(\boldsymbol{\beta};\boldsymbol{\xi}) \mid \boldsymbol{\beta}] \\
&= L_\epsilon(\boldsymbol{\beta})^2,
\end{aligned}$$

where $L_\epsilon(\boldsymbol{\beta};\boldsymbol{\xi}) = \sup_{\boldsymbol{\beta}'\in\mathcal{B},\|\boldsymbol{\beta}'-\boldsymbol{\beta}\|\leqslant\epsilon} \|\boldsymbol{U}(\boldsymbol{\xi};\boldsymbol{\beta})\|$, $L_\epsilon(\boldsymbol{\beta}) = \mathbb{E}\left[L_\epsilon(\boldsymbol{\beta};\boldsymbol{\xi})^2 \mid \boldsymbol{\beta}\right]^{\frac{1}{2}}$. And $L_\epsilon(\boldsymbol{\beta}) < \infty$ for all $\boldsymbol{\beta} \in \mathcal{B}$ by Lemma 8 of Duchi & Ruan (2018).

So we have

$$\mathbb{E}[\boldsymbol{\epsilon}_{\gamma_t}(\boldsymbol{\xi}^{(t)};\boldsymbol{\beta}^{(t-1)}) \mid \mathcal{F}_{t-1}] = 0, \quad \mathbb{E}[\|\boldsymbol{\epsilon}_{\gamma_t}(\boldsymbol{\xi}^{(t)};\boldsymbol{\beta}^{(t-1)})\|^2 \mid \mathcal{F}_{t-1}] \leqslant L_\epsilon(\boldsymbol{\beta}^{(t-1)})^2, \tag{A.2}$$

since $\boldsymbol{\beta}^{(t-1)} \in \mathcal{F}_{t-1}$, and given $\boldsymbol{\beta}^{(t-1)}$, $\boldsymbol{\xi}^{(t)}$ is independent of $\boldsymbol{\xi}^{(s)}, s < t$. Note that the independence holds true for exact sampling; For MCMC sampling, independence can also be achieved for any precision after applying 'thinning' procedure.

Further since $\mathcal{B}$ is compact, there is a random variable B which is finite with probability 1, such that for $t \in \mathbb{N}$, $\|\boldsymbol{\beta}^{(t)}\| \leqslant B$. Together with step size condition in H5, we have

$$\sum_{t=1}^{\infty} \mathbb{E}\left[\gamma_t^2 \|\boldsymbol{\epsilon}_{\gamma_t}(\boldsymbol{\xi}^{(t)};\boldsymbol{\beta}^{(t-1)})\|^2 \mid \mathcal{F}_{t-1}\right] \leqslant \sum_{t=1}^{\infty} \gamma_t^2 \sup_{\|\boldsymbol{\beta}\|\leqslant B,\boldsymbol{\beta}\in\mathcal{B}} L_\epsilon(\boldsymbol{\beta})^2 \leqslant \infty.$$

Thus $\gamma_t \boldsymbol{\epsilon}_{\gamma_t}(\boldsymbol{\xi}^{(t)},\boldsymbol{\beta}^{(t-1)})$ is a l_2-summable martingale difference sequence adpated to $\mathcal{F}_{t-1}$. By standard martingale convergence result (e.g., Thm. 5.3.33, Dembo, 2016), we have with probability 1, $\lim_n \sum_{t=1}^n \gamma_t \boldsymbol{\epsilon}_{\gamma_t}(\boldsymbol{\xi}^{(t)},\boldsymbol{\beta}^{(t-1)})$ exist and is finite.

B Proof of Theorem 1

In Theorem 1, we establish the convergence of $\boldsymbol{\beta}^{(t)}$ to a stationary point $\boldsymbol{\beta}_\infty \in \mathcal{B}^*$ using differential inclusion techniques in Duchi & Ruan (2018). The proposed method can be viewed as a special case of the general stochastic method discussed in Duchi & Ruan (2018) with a few differences.

With additional assumptions, a similar convergence result can be derived. In what follows, we first show the linear interpolation process of our stochastic updates is asymptotically equivalent to a differential inclusion, by verifying that conditions of Theorem 2 in Duchi & Ruan (2018) hold for our case. Then, cluster points of any trajectory of the limiting differential inclusion are proved to be stationary points. Lastly, the convergence properties of our original sequence can be shown from the functional convergence.

First we define the linear interpolation of the iterates $\boldsymbol{\beta}^{(k)}$:

$$\boldsymbol{\beta}(t) = \boldsymbol{\beta}^{(k)} + \frac{t - t_k}{t_{k+1} - t_k}(\boldsymbol{\beta}^{(k+1)} - \boldsymbol{\beta}^{(k)}) \text{ and } y(t) = \mathbb{E}[\boldsymbol{U}_{\gamma_k}(\boldsymbol{\xi}^{(k)}; \boldsymbol{\beta}^{(k-1)}) \mid \boldsymbol{\beta}^{(k-1)}] \text{ for } t \in [t_k, t_{k+1}),$$

and $\boldsymbol{\beta}^t(\cdot) = \boldsymbol{\beta}(t + \cdot), t \in \mathbb{R}_+$ be the time-shifted process.

In order to use Theorem 2 of Duchi & Ruan (2018), which is a general functional convergence theorem, conditions (i)-(iv) of Theorem 2 need to be verified for our case. Firstly, the boundness condition (i) holds as $\mathcal{B}$ is compact given H1; Non-summable but square-summable steps size condition (ii) holds given H5; And we have verified (iii), which is the convergence of the summation of the weighted noise sequence, holds by Lemma 1; Lastly, condition (iv) holds similarly in our case for the close-value mapping $-\boldsymbol{U}(\boldsymbol{\beta}) - \mathcal{N}_{\mathcal{B}}(\boldsymbol{\beta})$ (see Lemma 10 in Duchi & Ruan (2018)), where $\boldsymbol{U}(\boldsymbol{\beta}) = \nabla h(\boldsymbol{\beta}) + \partial g(\boldsymbol{\beta})$ and $\mathcal{N}_{\mathcal{B}}(\boldsymbol{\beta}) = \{\boldsymbol{v} \in \mathbb{R}^p : \langle \boldsymbol{v}, \boldsymbol{\beta}' - \boldsymbol{\beta} \rangle, \text{for all } \boldsymbol{\beta}' \in \mathcal{B}\}$ is the normal cone for $\mathcal{B}$ at $\boldsymbol{\beta}$.

Based on Theorem 2 and Theorem 3 of Duchi & Ruan (2018), for any sequences $\{\tau_k\}_{k=1}^\infty$, the function sequence $\boldsymbol{\beta}^{\tau_k}(\cdot)$ is relatively compact in $\mathcal{C}(\mathbb{R}^+, \mathbb{R}^p)$ and for any $\tau_k \to \infty$, any

limit point of $\{\boldsymbol{\beta}^{\tau_k}(\cdot)\}$ in $\mathcal{C}(\mathbb{R}^+, \mathbb{R}^p)$ satisfies

$$\bar{\boldsymbol{\beta}}(t) = \bar{\boldsymbol{\beta}}(0) + \int_0^t y(\tau) d\tau, \text{ where } y(\tau) \in -\boldsymbol{U}(\boldsymbol{\beta}(\tau)) - \mathcal{N}_{\mathcal{B}}(\boldsymbol{\beta}(\tau)).$$

So the sample path of our algorithm is asymptotically equivalent to the differential inclusion

$$\dot{\boldsymbol{\beta}} \in -\boldsymbol{U}(\boldsymbol{\beta}) - \boldsymbol{N}_{\mathcal{B}}(\boldsymbol{\beta}). \tag{B.1}$$

and the converged differential inclusion have uniqueness and convergence properties (see Theorem 4 of Duchi & Ruan (2018)).

Finally, according to Theorem 1 of Duchi & Ruan (2018), with probability 1,

$$[\liminf_t F(\boldsymbol{\beta}^{(t)}), \limsup_t F(\boldsymbol{\beta}^{(t)})] \subset F(\mathcal{B}^*).$$

Consequently, given assumption H1, $\mathcal{B}$ is compact and $\mathcal{B}^*$ contains finite points, we have the objective value $F(\boldsymbol{\beta}^{(t)})$ converges and all cluster points of the sequence $\{\boldsymbol{\beta}^{(t)}\}$ belong to $\mathcal{B}^*$.

By further assumption that different stationary points in $\mathcal{B}^*$ have different objective values, we have $\boldsymbol{\beta}^{(t)}$ converges to a stationary point in $\mathcal{B}^*$, with probability 1.

C Proof of Theorem 2

Follow the proofs in Section 6 of Atchadé et al. (2017), we first prove several lemmas, then prove Theorem 2.

Lemma C.1. *If g is convex and Lipschitz on $\mathcal{B}_1$ with Lipschitz constant K, or $g = I_{\mathcal{B}}(\cdot)$. For $\boldsymbol{\beta}, \boldsymbol{\beta}' \in \mathcal{B}_1$, any $\gamma > 0$, and diagonal matrix $\mathbf{D}$ with diagonal entries $\delta_i \in [c_1, c_2], c_2 \geqslant c_1 > 0$, the following conditions hold.*

(i) $g\left(\operatorname{Prox}_{\gamma,g}^{\mathbf{D}}(\boldsymbol{\beta})\right) - g\left(\boldsymbol{\beta}'\right) \leqslant -\frac{1}{\gamma}\left\langle \operatorname{Prox}_{\gamma,g}^{\mathbf{D}}(\boldsymbol{\beta}) - \boldsymbol{\beta}', \operatorname{Prox}_{\gamma,g}^{\mathbf{D}}(\boldsymbol{\beta}) - \boldsymbol{\beta}\right\rangle_{\mathbf{D}}.$

(ii) $\left\|\operatorname{Prox}_{\gamma,g}^{\mathbf{D}}(\boldsymbol{\beta}) - \operatorname{Prox}_{\gamma,g}^{\mathbf{D}}\left(\boldsymbol{\beta}'\right)\right\|_{\mathbf{D}}^2 + \left\|\left(\operatorname{Prox}_{\gamma,g}^{\mathbf{D}}(\boldsymbol{\beta}) - \boldsymbol{\beta}\right) - \left(\operatorname{Prox}_{\gamma,g}^{\mathbf{D}}\left(\boldsymbol{\beta}'\right) - \boldsymbol{\beta}'\right)\right\|_{\mathbf{D}}^2 \leqslant \left\|\boldsymbol{\beta} - \boldsymbol{\beta}'\right\|_{\mathbf{D}}^2.$

(iii) $\sup_{\gamma \in (0, c_1/L]} \sup_{\boldsymbol{\beta} \in \mathcal{B}_1} \gamma^{-1} \left\| \mathrm{Prox}^{\mathbf{D}}_{\gamma,g}(\boldsymbol{\beta}) - \boldsymbol{\beta} \right\| < \infty$.

Proof of Lemma C.1.

If $g = I_{\mathcal{B}}(\cdot)$, then for $\boldsymbol{\beta} \in \mathcal{B}_1 \subset \mathcal{B}$, $\mathrm{Prox}^{\mathbf{D}}_{\gamma,g}(\boldsymbol{\beta}) = \boldsymbol{\beta}$, so (i)-(iii) hold.

If g is Lipschitz (thus lower semi-continuous) and convex, given $\boldsymbol{\beta}, \boldsymbol{\beta}' \in \mathcal{B}_1$, $\gamma > 0$,

Let $\boldsymbol{p} = \mathrm{Prox}^{\mathbf{D}}_{\gamma,g}(\boldsymbol{\beta}) = \underset{\mathbf{x} \in \mathbb{R}^n}{\arg\min} \left\{ g(\mathbf{x}) + \frac{1}{2\gamma} \|\mathbf{x} - \boldsymbol{\beta}\|^2_{\mathbf{D}} \right\}$, set $\boldsymbol{p}_\alpha = \alpha \boldsymbol{\beta}' + (1-\alpha)\boldsymbol{p}$, for $\alpha \in (0,1)$. We have

$$g(\boldsymbol{p}) + \frac{1}{2\gamma}\|\boldsymbol{p} - \boldsymbol{\beta}\|^2_{\mathbf{D}} \leqslant g(\boldsymbol{p}_\alpha) + \frac{1}{2\gamma}\|\boldsymbol{p}_\alpha - \boldsymbol{\beta}\|^2_{\mathbf{D}}$$

Due to the convexity of g,

$$\begin{aligned} g(\boldsymbol{p}) &\leqslant \alpha g(\boldsymbol{\beta}') + (1-\alpha) g(\boldsymbol{p}) + \frac{1}{2\gamma}\|\alpha\boldsymbol{\beta}' - \alpha\boldsymbol{p} + \boldsymbol{p} - \boldsymbol{\beta}\|^2_{\mathbf{D}} - \frac{1}{2\gamma}\|\boldsymbol{p} - \boldsymbol{\beta}\|^2_{\mathbf{D}} \\ &\leqslant \alpha g(\boldsymbol{\beta}') + (1-\alpha) g(\boldsymbol{p}) - \frac{\alpha}{\gamma}\langle \boldsymbol{p} - \boldsymbol{\beta}', \boldsymbol{p} - \boldsymbol{\beta}\rangle_{\mathbf{D}} + \frac{\alpha^2}{2\gamma}\|\boldsymbol{\beta}' - \boldsymbol{p}\|^2_{\mathbf{D}}. \end{aligned}$$

So

$$g(\boldsymbol{p}) - g(\boldsymbol{\beta}') \leqslant -\frac{1}{\gamma}\langle \boldsymbol{p} - \boldsymbol{\beta}', \boldsymbol{p} - \boldsymbol{\beta}\rangle_{\mathbf{D}} + \frac{\alpha}{2\gamma}\|\boldsymbol{\beta}' - \boldsymbol{p}\|^2_{\mathbf{D}}$$

Let $\alpha \downarrow 0$, we have the desired inequality (i).

Further let $\boldsymbol{q} = \mathrm{Prox}^{\mathbf{D}}_{\gamma,g}(\boldsymbol{\beta}')$, by (i), we have

$$\begin{aligned} g(\boldsymbol{p}) + \frac{1}{\gamma}\langle \boldsymbol{p} - \boldsymbol{q}, \boldsymbol{p} - \boldsymbol{\beta}\rangle_{\mathbf{D}} &\leqslant g(\boldsymbol{q}) \\ g(\boldsymbol{q}) + \frac{1}{\gamma}\langle \boldsymbol{q} - \boldsymbol{p}, \boldsymbol{q} - \boldsymbol{\beta}'\rangle_{\mathbf{D}} &\leqslant g(\boldsymbol{p}) \end{aligned}$$

So

$$0 \leqslant \langle \boldsymbol{p} - \boldsymbol{q}, \boldsymbol{\beta} - \boldsymbol{\beta}' - \boldsymbol{p} + \boldsymbol{q})\rangle_{\mathbf{D}},$$

and

$$\|\boldsymbol{p}-\boldsymbol{q}\|_{\mathbf{D}}^2 \leqslant \langle \boldsymbol{p}-\boldsymbol{q}, \boldsymbol{\beta}-\boldsymbol{\beta}'\rangle_{\mathbf{D}}$$

$$\|(\boldsymbol{p}-\boldsymbol{\beta})-(\boldsymbol{q}-\boldsymbol{\beta}')\|_{\mathbf{D}}^2 \leqslant \langle (\boldsymbol{\beta}-\boldsymbol{p})-(\boldsymbol{\beta}'-\boldsymbol{q}), \boldsymbol{\beta}-\boldsymbol{\beta}'\rangle_{\mathbf{D}}$$

Summation of the above two inequations yeilds (ii).

Given g is proper convex, Lipschitz on $\mathcal{B}_1$ with Lipschitz constant is K and (i), we have

$$0 \leqslant \gamma^{-1}\left\|\mathrm{Prox}_{\gamma,g}^{\mathbf{D}}(\boldsymbol{\beta})-\boldsymbol{\beta}\right\|_{\mathbf{D}}^2 \leqslant g(\boldsymbol{\beta})-g(\mathrm{Prox}_{\gamma,g}^{\mathbf{D}}(\boldsymbol{\beta})) \leqslant K\left\|\mathrm{Prox}_{\gamma,g}^{\mathbf{D}}(\boldsymbol{\beta})-\boldsymbol{\beta}\right\|_{\mathbf{D}}.$$

Thus (iii) holds.

□

Lemma C.2. *Assume H7 and* $\gamma \in (0, c_1/L]$, *for* $\boldsymbol{\beta}, \boldsymbol{\beta}', \boldsymbol{\xi} \in \mathcal{B}_1$,

$$\begin{aligned}-2\gamma\left(F\left(\mathrm{Prox}_{\gamma,g}^{\mathbf{D}}(\boldsymbol{\beta})\right)-F\left(\boldsymbol{\beta}'\right)\right) \geqslant{}& \left\|\mathrm{Prox}_{\gamma,g}^{\mathbf{D}}(\boldsymbol{\beta})-\boldsymbol{\beta}'\right\|_{\mathbf{D}}^2\\ &+2\left\langle \mathrm{Prox}_{\gamma,g}^{\mathbf{D}}(\boldsymbol{\beta})-\boldsymbol{\beta}', \boldsymbol{\xi}-\gamma\mathbf{D}^{-1}\nabla h(\boldsymbol{\xi})-\boldsymbol{\beta}\right\rangle_{\mathbf{D}}-\|\boldsymbol{\beta}'-\boldsymbol{\xi}\|_{\mathbf{D}}^2\end{aligned} \tag{C.1}$$

Proof of Lemma C.2.

Using descent lemma of Lipschitz function ∇h, for any $\gamma^{-1} \geqslant L/c_1$,

$$h(\boldsymbol{p})-h(\boldsymbol{\xi}) \leqslant \left\langle \mathbf{D}^{-1}\nabla h(\boldsymbol{\xi}), \boldsymbol{p}-\boldsymbol{\xi}\right\rangle_{\mathbf{D}}+\frac{1}{2\gamma}\|\boldsymbol{p}-\boldsymbol{\xi}\|_{\mathbf{D}}^2$$

Since h is convex, so $h(\boldsymbol{\xi})+\langle \nabla h(\boldsymbol{\xi}), \boldsymbol{\beta}'-\boldsymbol{\xi}\rangle \leqslant h(\boldsymbol{\beta}')$,

$$h(\boldsymbol{p})-h(\boldsymbol{\beta}') \leqslant \left\langle \mathbf{D}^{-1}\nabla h(\boldsymbol{\xi}), \boldsymbol{p}-\boldsymbol{\beta}'\right\rangle_{\mathbf{D}}+\frac{1}{2\gamma}\|\boldsymbol{p}-\boldsymbol{\xi}\|_{\mathbf{D}}^2$$

And

$$g(\boldsymbol{p})-g(\boldsymbol{\beta}') \leqslant -\frac{1}{\gamma}\langle \boldsymbol{p}-\boldsymbol{\beta}', \boldsymbol{p}-\boldsymbol{\beta}\rangle_{\mathbf{D}}$$

Summation of the above two, we have,

$$F(\boldsymbol{p}) - F(\boldsymbol{\beta}') \leqslant -\frac{1}{\gamma}\left\langle \boldsymbol{p} - \boldsymbol{\beta}', \boldsymbol{\xi} - \gamma \mathbf{D}^{-1}\nabla h(\boldsymbol{\xi}) - \boldsymbol{\beta}\right\rangle_{\mathbf{D}} + \frac{1}{2\gamma}\|\boldsymbol{\beta}' - \boldsymbol{\xi}\|_{\mathbf{D}}^2 - \frac{1}{2\gamma}\|\boldsymbol{p} - \boldsymbol{\beta}'\|_{\mathbf{D}}^2$$

□

Lemma C.3. *Let*

$$\begin{aligned} T_\gamma(\boldsymbol{\beta}) &= \operatorname{Prox}_{\gamma,g}^{\mathbf{D}}(\boldsymbol{\beta} - \gamma \mathbf{D}^{-1}\nabla h(\boldsymbol{\beta})), \\ S_\gamma(\boldsymbol{\beta}) &= \operatorname{Prox}_{\gamma,g}^{\mathbf{D}}(\boldsymbol{\beta} - \gamma \mathbf{D}^{-1}\mathbf{G}_{\boldsymbol{\beta}}(\boldsymbol{\xi})), \\ \boldsymbol{\eta} &= \mathbf{D}^{-1}\mathbf{G}_{\boldsymbol{\beta}}(\boldsymbol{\xi}) - \mathbf{D}^{-1}\nabla h(\boldsymbol{\beta}). \end{aligned}$$

Then for $\boldsymbol{\beta} \in \mathcal{B}_1$, and $\gamma > 0$,

$$\|T_\gamma(\boldsymbol{\beta}) - S_\gamma(\boldsymbol{\beta})\|_{\mathbf{D}} \leqslant \gamma\|\boldsymbol{\eta}\|_{\mathbf{D}} \tag{C.2}$$

Proof of Lemma C.3.

$$\begin{aligned} \|T_\gamma(\boldsymbol{\beta}) - S_\gamma(\boldsymbol{\beta})\|_{\mathbf{D}} &= \|\operatorname{Prox}_{\gamma,g}^{\mathbf{D}}(\boldsymbol{\beta} - \gamma \mathbf{D}^{-1}\nabla h(\boldsymbol{\beta})) - \operatorname{Prox}_{\gamma,g}^{\mathbf{D}}(\boldsymbol{\beta} - \gamma \mathbf{D}^{-1}\mathbf{G})\|_{\mathbf{D}} \\ &\leqslant \|\gamma \mathbf{D}^{-1}\mathbf{G} - \gamma \mathbf{D}^{-1}\nabla h(\boldsymbol{\beta})\|_{\mathbf{D}} \\ &\leqslant \gamma\|\boldsymbol{\eta}\|_{\mathbf{D}}, \end{aligned}$$

where the first inequality follows from Lemma C.1-(ii).

□

Lemma C.4. *Assume H4 and H8. Then* $\sup_t \mathbb{E}[W^p(\boldsymbol{\xi}_t)] < \infty$.

Proof of Lemma C.4. As the conditional distribution of $\boldsymbol{\xi}_t$ given $\mathcal{F}_{t-1}$ is $P_{\boldsymbol{\beta}^{(t-1)}}(\boldsymbol{\xi}_{t-1}, \cdot)$, so

$$\mathbb{E}[W^p(\boldsymbol{\xi}_t)] = \mathbb{E}[\mathbb{E}[W^p(\boldsymbol{\xi}_t)|\mathcal{F}_{t-1}]] = \mathbb{E}[P_{\boldsymbol{\beta}^{(t-1)}}W^p(\boldsymbol{\xi}_{t-1})] \leqslant \lambda\mathbb{E}[W^p(\boldsymbol{\xi}_{t-1})] + b.$$

And by induction the proof is concluded. $\square$

Lemma C.5. *Assume H1, H4, H7-(ii) and H8. There exist a constant C such that w.p.1, for all $t \geqslant 0$, $\|\boldsymbol{\eta}_t\| \leqslant CW(\boldsymbol{\xi}^{(t)})$.*

Proof of Lemma C.5. By definition,

$$\|\boldsymbol{\eta}_t\| = \|(\mathbf{D}^{(t)})^{-1}\mathbf{G}_{\boldsymbol{\beta}^{(t-1)}}(\boldsymbol{\xi}^{(t)}) - (\mathbf{D}^{(t)})^{-1}\nabla h(\boldsymbol{\beta}^{(t-1)})\| \leqslant \frac{1}{c_1}(\sup_{\boldsymbol{\beta}\in\mathcal{B}_1} |\mathbf{G}_{\boldsymbol{\beta}}|_W)W(\boldsymbol{\xi}^{(t)}) + \frac{1}{c_1}\sup_{\boldsymbol{\beta}\in\mathcal{B}_1} \|\nabla h(\boldsymbol{\beta})\|.$$

And the result follows as ∇h is Lipschitz and $W \geqslant 1$. $\square$

Lemma C.6. *Assume H1, H4, H5 and H8. If $a_t \geqslant 0$, for $t \geqslant 1$, there exist a constant C such that*

$$\left\| \sum_{t=1}^{n} a_t \|\boldsymbol{\eta}_t\|^2_{\mathbf{D}^{(t)}} \right\|_{L_2} \leqslant C \sum_{t=1}^{n} a_t \tag{C.3}$$

Proof of Lemma C.6. By Minkowski inequality,

$$\left\| \sum_{t=1}^{n} a_t \|\boldsymbol{\eta}_t\|^2_{\mathbf{D}^{(t)}} \right\|_{L_2} \leqslant C \sup_t \|\boldsymbol{\eta}_t\|^2_{L_4} \sum_{t=1}^{n} a_t \leqslant C \sum_{t=1}^{n} a_t,$$

as the supremum is finite based on Lemma C.4 and Lemma C.5. $\square$

Lemma C.7. *Assume H1, H4, and H6. Then*

$$\sup_{\gamma\in(0,c_1/L]} \sup_{\boldsymbol{\beta}\in\mathcal{B}_1} \|T_\gamma(\boldsymbol{\beta})\| < \infty. \tag{C.4}$$

If additional H7-(ii) holds, then there exist a constant C such that for any $\boldsymbol{\beta}, \boldsymbol{\beta}' \in \mathcal{B}_1$, $\gamma, \gamma' \in (0, c_1/L]$,

$$\|T_\gamma(\boldsymbol{\beta}) - T_{\gamma'}(\boldsymbol{\beta}')\| \leqslant C(\gamma + \gamma\prime + \|\boldsymbol{\beta} - \boldsymbol{\beta}'\|) \tag{C.5}$$

Proof of Lemma C.7. As $\boldsymbol{\beta}_\star = T_\gamma(\boldsymbol{\beta}_\star)$ for any $\gamma > 0$. And

$$
\begin{aligned}
\|T_\gamma(\boldsymbol{\beta}) - \boldsymbol{\beta}_\star\| &= \|T_\gamma(\boldsymbol{\beta}) - T_\gamma(\boldsymbol{\beta}_\star)\| \\
&= \|\mathrm{Prox}^{\mathbf{D}}_{\gamma,g}(\boldsymbol{\beta} - \gamma\mathbf{D}^{-1}\nabla h(\boldsymbol{\beta})) - \mathrm{Prox}^{\mathbf{D}}_{\gamma,g}(\boldsymbol{\beta}_\star - \gamma\mathbf{D}^{-1}\nabla h(\boldsymbol{\beta}_\star))\| \\
&\leqslant \frac{1}{c_1}\|\boldsymbol{\beta} - \gamma\mathbf{D}^{-1}\nabla h(\boldsymbol{\beta}) - \boldsymbol{\beta}_\star + \gamma\mathbf{D}^{-1}\nabla h(\boldsymbol{\beta}_\star)\|_{\mathbf{D}} \\
&\leqslant (1 + \frac{c_2}{c_1})\,(\|\boldsymbol{\beta}\| + \|\boldsymbol{\beta}_\star\|) < \infty,
\end{aligned}
$$

where the first and second inequality comes from Lipschitz property of $\mathrm{Prox}^{\mathbf{D}}_{\gamma,g}$ (see H7-(ii)) and ∇h, respectively. So we have (C.4) holds.

To prove (C.5), decompose $T_\gamma(\boldsymbol{\beta}) - T_{\gamma'}(\boldsymbol{\beta}') = T_\gamma(\boldsymbol{\beta}) - T_{\gamma'}(\boldsymbol{\beta}) + T_{\gamma'}(\boldsymbol{\beta}) - T_{\gamma'}(\boldsymbol{\beta}')$.

$$
\begin{aligned}
\|T_{\gamma'}(\boldsymbol{\beta}) - T_{\gamma'}(\boldsymbol{\beta}')\| &\leqslant \frac{1}{c_1}\|\boldsymbol{\beta} - \gamma'\mathbf{D}^{-1}\nabla h(\boldsymbol{\beta}) - \boldsymbol{\beta}' + \gamma'\mathbf{D}^{-1}\nabla h(\boldsymbol{\beta}')\|_{\mathbf{D}} \\
&\leqslant \frac{c_2}{c_1}\|\boldsymbol{\beta} - \boldsymbol{\beta}'\| + \frac{2\sup_{\boldsymbol{\beta}\in\mathcal{B}_1}\|\nabla h(\boldsymbol{\beta})\|}{c_1}\gamma' \\
&\leqslant C(\gamma' + \|\boldsymbol{\beta} - \boldsymbol{\beta}'\|).
\end{aligned}
$$

Since H6 and $\mathcal{B}_1$ is compact, $\sup_{\boldsymbol{\beta}\in\mathcal{B}_1}\|\nabla h(\boldsymbol{\beta})\| < \infty$.

$$
\begin{aligned}
\|T_\gamma(\boldsymbol{\beta}) - T_{\gamma'}(\boldsymbol{\beta})\| &= \|\mathrm{Prox}^{\mathbf{D}}_{\gamma,g}(\boldsymbol{\beta} - \gamma\mathbf{D}^{-1}\nabla h(\boldsymbol{\beta})) - \mathrm{Prox}_{\gamma',g}(\boldsymbol{\beta} - \gamma'\mathbf{D}^{-1}\nabla h(\boldsymbol{\beta}))\| \\
&= \|\mathrm{Prox}^{\mathbf{D}}_{\gamma,g}(\boldsymbol{\beta} - \gamma\mathbf{D}^{-1}\nabla h(\boldsymbol{\beta})) - \mathrm{Prox}^{\mathbf{D}}_{\gamma,g}(\boldsymbol{\beta})\| \\
&+ \|\mathrm{Prox}^{\mathbf{D}}_{\gamma',g}(\boldsymbol{\beta} - \gamma'\mathbf{D}^{-1}\nabla h(\boldsymbol{\beta})) - \mathrm{Prox}^{\mathbf{D}}_{\gamma',g}(\boldsymbol{\beta})\| \\
&+ \|\mathrm{Prox}^{\mathbf{D}}_{\gamma,g}(\boldsymbol{\beta}) - \mathrm{Prox}^{\mathbf{D}}_{\gamma',g}(\boldsymbol{\beta})\| \\
&\leqslant \frac{1}{c_1}\left(\sup_{\boldsymbol{\beta}\in\mathcal{B}_1}\|\nabla h(\boldsymbol{\beta})\|(\gamma + \gamma') + \|\mathrm{Prox}^{\mathbf{D}}_{\gamma,g}(\boldsymbol{\beta}) - \boldsymbol{\beta}\|_{\mathbf{D}} + \|\mathrm{Prox}^{\mathbf{D}}_{\gamma',g}(\boldsymbol{\beta}) - \boldsymbol{\beta}\|_{\mathbf{D}}\right) \\
&\leqslant \frac{1}{c_1}\left(\sup_{\boldsymbol{\beta}\in\mathcal{B}_1}\|\nabla h(\boldsymbol{\beta})\| + c_2\sup_{\gamma\in(0,c_1/L]}\sup_{\boldsymbol{\beta}\in\mathcal{B}_1}\|\mathrm{Prox}_{\gamma,g}(\boldsymbol{\beta}) - \boldsymbol{\beta}\|\right)(\gamma + \gamma') \leqslant C(\gamma + \gamma').
\end{aligned}
$$

The above inequality follows from assumption H7-(ii). □

Proof of Theorem 2.

By assumption $\boldsymbol{\beta}_\star \in \mathcal{B}_1$, and $\boldsymbol{\beta}_\star = \arg\min_{\boldsymbol{\beta}\in\mathcal{B}_1} F(\boldsymbol{\beta}) := \min F$. Apply (C.1) with $\boldsymbol{\beta} \leftarrow \boldsymbol{\beta}^{(t)} - \gamma_{t+1}\left(\mathbf{D}^{(t+1)}\right)^{-1}\mathbf{G}_{\boldsymbol{\beta}^{(t)}}(\boldsymbol{\xi}^{(t+1)})$, $\boldsymbol{\xi} \leftarrow \boldsymbol{\beta}^{(t)}$, $\boldsymbol{\beta}' \leftarrow \boldsymbol{\beta}_\star, \gamma \leftarrow \gamma_{t+1}, \mathbf{D} \leftarrow \mathbf{D}^{(t+1)}$, we have

$$\|\boldsymbol{\beta}^{(t+1)}-\boldsymbol{\beta}_\star\|^2_{\mathbf{D}^{(t+1)}} \leqslant \|\boldsymbol{\beta}^{(t)}-\boldsymbol{\beta}_\star\|^2_{\mathbf{D}^{(t+1)}} - 2\gamma_{t+1}\left(F(\boldsymbol{\beta}^{(t+1)}) - F(\boldsymbol{\beta}_\star)\right) - 2\gamma_{t+1}\left\langle \boldsymbol{\beta}^{(t+1)} - \boldsymbol{\beta}_\star, \boldsymbol{\eta}_{t+1}\right\rangle_{\mathbf{D}^{(t+1)}}. \tag{C.6}$$

By rearranging (C.6), we have

$$\begin{aligned}
F(\boldsymbol{\beta}^{(t+1)}) - F(\boldsymbol{\beta}_\star) &\leqslant \frac{1}{2\gamma_{t+1}}\left(\|\boldsymbol{\beta}^{(t)} - \boldsymbol{\beta}_\star\|^2_{\mathbf{D}^{(t+1)}} - \|\boldsymbol{\beta}^{(t+1)} - \boldsymbol{\beta}_\star\|^2_{\mathbf{D}^{(t+1)}}\right) - \left\langle \boldsymbol{\beta}^{(t+1)} - \boldsymbol{\beta}_\star, \boldsymbol{\eta}_{t+1}\right\rangle_{\mathbf{D}^{(t+1)}} \\
&\leqslant \frac{1}{2}\left(\frac{1}{\gamma_{t+1}} - \frac{1}{\gamma_t}\right)\|\boldsymbol{\beta}^{(t)} - \boldsymbol{\beta}_\star\|^2_{\mathbf{D}^{(t+1)}} - \frac{1}{2\gamma_{t+1}}\|\boldsymbol{\beta}^{(t+1)} - \boldsymbol{\beta}_\star\|^2_{\mathbf{D}^{(t+1)}} \\
&\quad + \frac{1}{2\gamma_t}\|\boldsymbol{\beta}^{(t)} - \boldsymbol{\beta}_\star\|^2_{\mathbf{D}^{(t+1)}} - \left\langle \boldsymbol{\beta}^{(t+1)} - \boldsymbol{\beta}_\star, \boldsymbol{\eta}_{t+1}\right\rangle_{\mathbf{D}^{(t+1)}}
\end{aligned}$$

Sum from $t = 0, \ldots, n-1$, and decompose

$$\langle \boldsymbol{\beta}^{(t)} - \boldsymbol{\beta}_\star, \boldsymbol{\eta}_t\rangle_{\mathbf{D}^{(t)}} = \left\langle \boldsymbol{\beta}^{(t)} - T_{\gamma_t}\left(\boldsymbol{\beta}^{(t-1)}\right), \boldsymbol{\eta}_t\right\rangle_{\mathbf{D}^{(t)}} + \left\langle T_{\gamma_t}\left(\boldsymbol{\beta}^{(t-1)}\right) - \boldsymbol{\beta}_\star, \boldsymbol{\eta}_t\right\rangle_{\mathbf{D}^{(t)}}.$$

By (C.2), we have $\left|\left\langle \boldsymbol{\beta}^{(t)} - T_{\gamma_t}\left(\boldsymbol{\beta}^{(t-1)}\right), \boldsymbol{\eta}_t\right\rangle_{\mathbf{D}^{(t)}}\right| \leqslant \gamma_t\|\boldsymbol{\eta}_t\|^2_{\mathbf{D}^{(t)}}$, so

$$\begin{aligned}
\sum_{t=1}^n \left(F(\boldsymbol{\beta}^{(t)}) - \min F\right) &\leqslant \sum_{t=1}^n \frac{1}{2}\left(\frac{1}{\gamma_t} - \frac{1}{\gamma_{t-1}}\right)\|\boldsymbol{\beta}^{(t-1)} - \boldsymbol{\beta}_\star\|^2_{\mathbf{D}^{(t)}} + \frac{1}{2\gamma_0}\|\boldsymbol{\beta}^{(0)} - \boldsymbol{\beta}_\star\|^2_{\mathbf{D}^{(1)}} \\
&\quad + \sum_{t=1}^{n-1}\frac{1}{2\gamma_t}\|\boldsymbol{\beta}^{(t)} - \boldsymbol{\beta}_\star\|^2_{\mathbf{D}^{(t+1)}-\mathbf{D}^{(t)}} - \sum_{t=1}^n \left\langle T_{\gamma_t}\left(\boldsymbol{\beta}^{(t-1)}\right) - \boldsymbol{\beta}_\star, \eta_t\right\rangle_{\mathbf{D}^{(t)}} + \sum_{t=1}^n \gamma_t\|\boldsymbol{\eta}_t\|^2_{\mathbf{D}^{(t)}}
\end{aligned} \tag{C.7}$$

Under the assumptions H6, the function F is convex so that

$$F(\bar{\boldsymbol{\beta}}_n) \leqslant \frac{1}{n}\sum_{t=1}^n F(\boldsymbol{\beta}^{(t)}). \tag{C.8}$$

Denote $\|\cdot\|_{L_2} = (\mathbb{E}\|\cdot\|^2)^{1/2}$. By (C.7) and Minkowski inequality, we have there exists a

constant $C > 0$, such that

$$\|F(\bar{\boldsymbol{\beta}}_n) - \min F\|_{L_2} \leqslant \frac{C}{n}\Bigg(\sum_{t=1}^{n} \left| \frac{1}{\gamma_t} - \frac{1}{\gamma_{t-1}} \right| + \frac{1}{\gamma_0} + \sum_{t=1}^{n-1} \frac{1}{\gamma_t} \left\| \mathbf{D}^{(t+1)} - \mathbf{D}^{(t)} \right\|_{L_2} + \left\| \sum_{t=1}^{n} \left\langle T_{\gamma_t}(\boldsymbol{\beta}^{(t)}), \boldsymbol{\eta}_t \right\rangle_{\mathbf{D}^{(t)}} \right\|_{L_2} + \left\| \sum_{t=1}^{n} \langle \boldsymbol{\beta}_\star, \boldsymbol{\eta}_t \rangle_{\mathbf{D}^{(t)}} \right\|_{L_2} + \left\| \sum_{t=1}^{n} \gamma_t \|\boldsymbol{\eta}_t\|^2_{\mathbf{D}^{(t)}} \right\|_{L_2} \Bigg).$$

By assumption, we assume $\gamma_t = Ct^{-\alpha}, \alpha \in (1/2, 1]$,

$$\sum_{t=1}^{n} \left| \frac{1}{\gamma_t} - \frac{1}{\gamma_{t-1}} \right| = O(n^\alpha), \quad \sum_{t=1}^{n-1} \frac{1}{\gamma_t} \left\| \mathbf{D}^{(t+1)} - \mathbf{D}^{(t)} \right\|_{L_2} = O(n^\alpha)$$

Apply $a_t = \gamma_t$, in Lemma C.6

$$\left\| \sum_{t=1}^{n} \gamma_t \|\boldsymbol{\eta}_t\|^2_{\mathbf{D}^{(t-1)}} \right\|_{L_2} \leqslant C \sum_{t=1}^{n} \gamma_t, \tag{C.9}$$

and $\sum_{t=1}^{n} \gamma_t = O(n^{1-\alpha})$ for $\alpha \in (1/2, 1)$, and $\sum_{t=1}^{n} \gamma_t = O(\ln n)$ for $\alpha = 1$.

When $\boldsymbol{\xi}^{(t)}$ are sampled exactly, i.e., unbiased case, combine Lemma C.7 and Proposition 18 of Atchadé et al. (2017), there exists a constant C such that

$$\left\| \sum_{t=0}^{n} \left\langle \mathbf{A}_{\gamma_{t+1}}(\boldsymbol{\beta}^{(t)}), \boldsymbol{\eta}_t \right\rangle_{\mathbf{D}^{(t)}} \right\|_{L_2} \leqslant C\sqrt{n}.$$

Similarly, for the case of biased approximation, combine Lemma C.7, and Proposition 19 of Atchadé et al. (2017), there exists a constant C such that

$$\left\| \sum_{t=0}^{n} \left\langle \mathbf{A}_{\gamma_{t+1}}(\boldsymbol{\beta}^{(t)}) \boldsymbol{\eta}_t \right\rangle_{\mathbf{D}^{(t)}} \right\|_{L_2} \leqslant C \left(1 + \sqrt{n} + \sum_{t=0}^{n} \gamma_t \right).$$

In both cases, let $\mathbf{A}_{\gamma_t}(\boldsymbol{\beta}^{(t-1)}) = T_{\gamma_t}(\boldsymbol{\beta}^{(t-1)})$ and $\mathbf{A}_{\gamma_t}(\boldsymbol{\beta}^{(t-1)}) = I$, we have

$$\left\| \sum_{t=1}^{n} \left\langle T_{\gamma_t}(\boldsymbol{\beta}^{(t-1)}), \boldsymbol{\eta}_t \right\rangle_{\mathbf{D}^{(t-1)}} \right\|_{L_2} = O(\sqrt{n}) \text{ and } \left\| \sum_{t=1}^{n} \langle \boldsymbol{\beta}_\star, \boldsymbol{\eta}_t \rangle_{\mathbf{D}^{(t-1)}} \right\|_{L_2} = O(\sqrt{n})$$

Combine the above results and as h is strongly convex, so there exist a $\mu > 0$, such that $F(\bar{\boldsymbol{\beta}}_n) - F(\boldsymbol{\beta}_\star) \geqslant \frac{\mu}{2}\|\bar{\boldsymbol{\beta}}_n - \boldsymbol{\beta}_\star\|^2$, so we have

$$\mathbb{E}\|\bar{\boldsymbol{\beta}}_n - \boldsymbol{\beta}_\star\|^2 \leqslant \left(\mathbb{E}\|\bar{\boldsymbol{\beta}}_n - \boldsymbol{\beta}_\star\|^4\right)^{1/2} \leqslant C\|F(\bar{\boldsymbol{\beta}}_n) - \min F\|_{L_2} \leqslant Cn^{\alpha-1}.$$

As $\alpha \in (1/2, 1]$, by choosing $\alpha = 1/2 + \epsilon, \epsilon > 0$, we have the lowest bound $Cn^{-\frac{1}{2}+\epsilon}$. □

D Additional Simulation Results

We provide an additional simulation study to (1) assess the estimation of the asymptotic variances of parameter estimates and (2) assess the point estimation of the covariance between latent variables. We consider a similar confirmatory IFA setting as in the simulation study I, with two factors, twenty items (i.e., $K = 2$, $J = 20$), and the same design matrix $\mathbf{Q}$. The intercept parameters and non-zero loading parameters are drawn i.i.d. from the standard normal and a uniform distribution over the interval $(0.5, 1.5)$, respectively. The variances of two factors are set to be 1 and the covariance is set to be 0.4. For each of the three sample sizes $N = 1000, 2000, 4000$, 50 independent datasets are generated. We then apply the proposed USP method with 1000 burn-in size, and 4000 total iterations. Note that we use a larger burn-in size and a larger number of iterations here to ensure accurate computation of the asymptotic variances, because they tend to be more difficult to compute than the point estimates. The results from the UPS algorithm are compared with those from a standard EM algorithm that uses 31 quadrature points for each dimension.

We approximate the observed Fisher information matrix using the approach given in Remark 8. Based on the approximated Fisher information matrix, we obtain the standard errors of parameter estimates. The obtained standard errors are compared with those given by the EM algorithm. The results are given in Figure D.1. Each panel of Figure D.1 corresponds to a combination of a sample size and a type of parameters (loadings/intercepts/covariance). For each dataset and each parameter, we obtain the standard errors of the parameter estimate from the UPS and EM algorithms, respectively. These standard errors are shown as a point in the scatter plot, where the x-axis gives the standard error from the EM algorithm and the y-axis gives the standard error from

the USP method. As we can see, all the points concentrate along the diagonal line, suggesting that the standard errors from the two algorithms are very close to each other.

We further assess the estimation of the covariance between the latent variables. The results are given in Figure D.2. For each sample size, we compute the squared difference between the estimate given by the USP algorithm and the true value ($\sigma_{12} = 0.4$) and visualize the squared errors from the 50 datasets using a box plot. We see that all the squared errors are quite small and they decrease when the sample size increases.

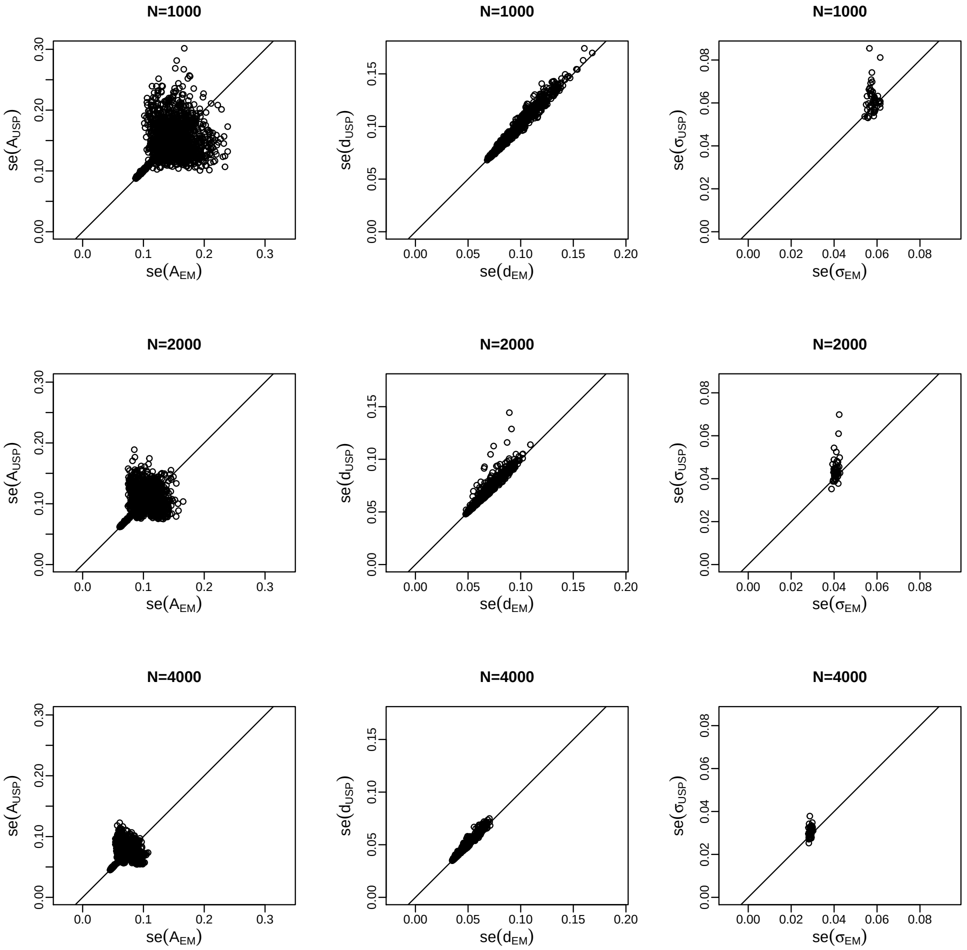

Figure D.1: Scatter plots of standard error estimates for loading parameters $\mathbf{A}$, intercept parameters $\boldsymbol{d}$, and correlation parameter σ, from the EM method and the USP method under different sample sizes. The x-axis and y-axis represent standard error estimates from the EM and the USP method respectively. Each row corresponds to one sample size and each column corresponds to one type of parameter.

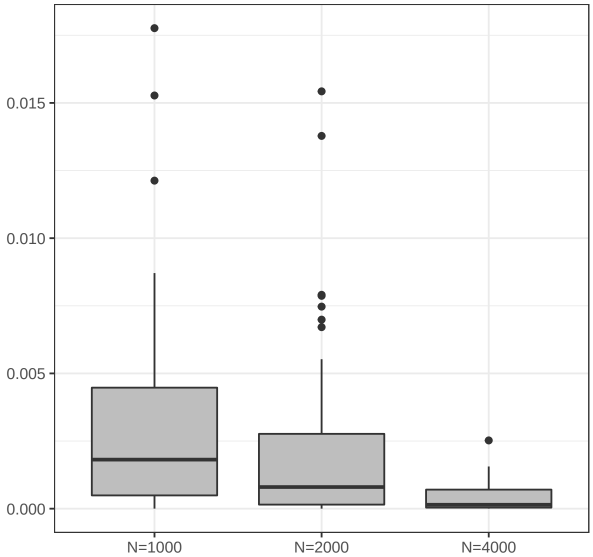

Figure D.2: Box plots of squared errors for estimated correlation parameter σ_{12} from the USP method.

References

Atchadé, Y. F., Fort, G., & Moulines, E. (2017). On perturbed proximal gradient algorithms. *The Journal of Machine Learning Research*, *18*, 310–342.

Dembo, A. (2016). *Probability theory: Stat310/math230, lecture notes.* Stanford, CA. Retrieved from `http://statweb.stanford.edu/~adembo/stat-310b/lnotes.pdf` (Last visited on 2020/07/16)

Duchi, J. C., & Ruan, F. (2018). Stochastic methods for composite and weakly convex optimization problems. *SIAM Journal on Optimization*, *28*, 3229–3259.
